# Letter to the editor in response to "Agreement between continuous and intermittent pulmonary artery thermodilution for cardiac output measurement in perioperative and intensive care medicine: a systematic review and meta-analysis"

**DOI:** 10.1186/s13054-021-03613-6

**Published:** 2021-06-21

**Authors:** Fellery de Lange, Inge T. Bootsma, E. Christiaan Boerma

**Affiliations:** Department of Intensive Care, Medical Center Leeuwarden, Henri Dunantweg 2, P.O. Box 888, 8901 Leeuwarden, The Netherlands

**Dear editor,**

It is with great interest that we have read the recent article on agreement between intermittent and continuous pulmonary artery (PA) thermodilution measurements by Kouz et al. [1]. However, we feel the conclusions are hampered by incorrect assumptions, which we would like to address.

First, the authors consider intermittent cardiac output (CO) measurements the Gold Standard. Although historically correct, such approach is clearly debatable. Intermittent PA CO measurements are by definition dependent on injected volume, temperature, and rate. Inadequate timing during the respiratory cycle further influences its accuracy. All of us who have regularly done this by hand, know how variable these CO measurements are, even when executed by one well-trained person within the shortest possible timeframe [2]. Generally, a ‘single’ intermittent CO measurement represents an average of 3 to 5 individual measurements. Continuous CO measurements (CCO) are based on a thermistor, which continuously measures changes in blood temperature caused by the thermal filament, during the entire respiratory cycle in an on–off fashion. The high sampling rate at random time points in the ventilatory cycle allows for an operator-independent detection of smaller variations in CO, as well as good performance over a wide range of CO and blood temperatures [3]. With this in mind, we do not understand why the authors consider intermittent CO monitoring as the Gold Standard in comparison to CCO monitoring. This should definitely be reversed; an earlier method is not by definition normative.

Secondly, the authors state that “continuous” should actually be “semi-continuous” and that there is a delay up to several minutes. This is partially incorrect, since the thermal signal is measured continuously, but updated every 30 to 60 s as ‘STAT CCO’. In case of major changes an update of the CO will be displayed after 270 s [4]. The claim that “This time delay may become relevant when hemodynamics change rapidly, e.g., during dynamic tests such as passive leg raising and during therapeutic interventions such as fluid or vasopressor administration” misses the point that with intermittent CO measurements detection of these swift changes is also impossible. To measure the swift CO change during a passive leg raising test, measurement by pulse contour analysis is mandatory. To evaluate all other hemodynamic interventions, including the longer lasting effect of fluid administration and vasopressors, CCO measurement is far superior to intermittent CO measurement. CCO measurements are more reliable, faster, operator- and timing independent and hence the clinical method of reference [5].

## Data Availability

Not applicable.
