# Supplementary material for: Letter to the editor in response to "Agreement between continuous and intermittent pulmonary artery thermodilution for cardiac output measurement in perioperative and intensive care medicine: a systematic review and meta-analysis"
Source: Crit Care. 2021 Jun 21;25:215. doi: 10.1186/s13054-021-03613-6 (PMC8215795; doi:10.1186/s13054-021-03613-6)
Supplement: Related texts

# Reply: Author’s response

**Authors:** Karim Kouz, Frederic Michard, Alina Bergholz, Christina Vokuhl, Luisa Briesenick, Phillip Hoppe, Moritz Flick, Gerhard Schön, Bernd Saugel

We thank de Lange et al. for their interest in our meta-analysis of clinical studies comparing continuous with intermittent pulmonary artery thermodilution cardiac output measurements [1]. De Lange et al. express concerns whether intermittent pulmonary artery thermodilution can be considered as the clinical reference cardiac output monitoring method. In our meta-analysis, we do not advocate for defining  intermittent pulmonary artery thermodilution as the reference method—but we used it as the reference method because most of the studies we included in the analysis did. We systematically investigated whether cardiac output measurements obtained by continuous pulmonary artery thermodilution were interchangeable with those obtained by intermittent pulmonary artery thermodilution. Irrespective of which of the two methods one considers the reference method, the conclusion about their interchangeability is the same.

The authors further stimulate a discussion on whether automated continuous pulmonary artery thermodilution is actually a *continuous—*or rather a *semi-continuous—*cardiac output monitoring method. Although its usually called “continuous” pulmonary artery thermodilution we would prefer calling it *automated semi-continuous* pulmonary artery thermodilution*.* For automated semi-continuous pulmonary artery thermodilution a filament attached to the catheter heats up the blood in a random sequence (in contrast to cold fluid boluses used for intermittent pulmonary artery thermodilution) [6, 7]. The magnitude of changes in blood temperature is much smaller when induced by a filament heating up the blood in a random sequence than by a cold fluid bolus. Multiple consecutive measurements are averaged when using automated semi-continuous pulmonary artery thermodilution. Rapid changes in global hemodynamics may delay the stabilization period of the system and, therefore, the cardiac output is not displayed continuously in real-time, but rather with a time delay of up to a few minutes.
